# Resistance-gene minority variation and delayed microbiological response in rifampicin-resistant tuberculosis

**DOI:** 10.3389/fcimb.2026.1930268

**Published:** 2026-10-22

**Authors:** Qing Fang, Xiangchen Li, Yewei Lu, Dongliang Zhang, Fang Li, Junli Gao, Yang Che

**Affiliations:** 1 Departments of Pulmonary Medicine, The First Affiliated Hospital of Ningbo University, Ningbo, Zhejiang, China; 2 Cosmos Wisdom Biotech Co. Ltd, Hangzhou, Zhejiang, China; 3 Research and Innovation Center, Cosmos Wisdom Biotech Co. Ltd, Hangzhou, Zhejiang, China; 4 Institute of Tuberculosis Prevention and Control, Ningbo Municipal Center for Disease Control and Prevention, Ningbo, Zhejiang, China

**Keywords:** culture conversion, minority variants, multidrug-resistant tuberculosis, rifampicin-resistant tuberculosis, treatment outcome, whole-genome sequencing

## Abstract

**Introduction:**

Routine whole-genome sequencing (WGS) interpretation of *Mycobacterium tuberculosis* resistance focuses mainly on consensus-level mutations. Minority variants, quantified by allele frequency (AF), may indicate resistant subpopulations under drug pressure, but their association with treatment response in rifampicin-resistant or multidrug-resistant tuberculosis (RR/MDR-TB) remains unclear.

**Methods:**

We retrospectively analysed RR/MDR-TB patients treated in Ningbo, China, from 2021 to 2025, integrating clinical, phenotypic, treatment, microbiological, and WGS data. Minority resistance-associated variants (RAVs) were defined as low-frequency variants matching WHO V2 mutation catalogue entries at AF ≥ 3%, with AF ≥ 5% defining the primary higher-frequency subset. Per-drug analyses excluded patients whose target genes had median depth <200×. Associations with microbiological response were assessed using covariate-adjusted logistic regression, with higher-frequency and treatment-matched variants examined secondarily.

**Results:**

We analysed 229 RR/MDR-TB patients with linked WGS, treatment, and microbiological follow-up data. Fixed RAVs (WHO V2 catalogue-annotated) were carried by 220/229 patients (median 3, IQR 2–5). Minority RAVs at AF ≥ 3% were detected in 13 patients. Overall minority-RAV burden was not clearly associated with microbiological response after adjustment. The clearest signal involved treatment-matched AF ≥ 5% minority RAVs, whose carriers had a higher 6-month culture-positivity rate than non-carriers (4/7 [57.1%] vs 40/198 [20.2%]; adjusted odds ratio [aOR] 7.21, 95% CI 1.38–43.68; *p* = 0.020; *q* = 0.034), with a modest AUC increase from 0.675 to 0.695. Six-month positivity was highest among patients with both high fixed RAV burden and treatment-matched AF ≥ 5% minority RAVs (4/5 [80.0%] vs 15/106 [14.2%] in the low-burden non-carrier reference; Fisher’s exact *p* = 0.003).

**Conclusion:**

Minority variants showed limited overall prognostic value, but higher-frequency treatment-matched variants provided a coherent signal for 6-month culture positivity when considered with fixed RAV burden. These exploratory findings support a cautious, treatment-aware review of selected minority variants and require external validation before clinical use.

## Introduction

Rifampicin-resistant and multidrug-resistant tuberculosis (RR/MDR-TB) remain major obstacles to tuberculosis control (Seddon et al., 2025). Recent World Health Organization (WHO) reports and treatment guidance emphasize that improved diagnostics, shorter all-oral regimens, and stronger drug-resistance surveillance are now central to RR/MDR-TB control, yet treatment response remains heterogeneous even among patients whose baseline resistance profile appears similar by conventional testing (World Health Organization, 2025a, 2025b). Although culture conversion is not a perfect surrogate for relapse-free cure, delayed sputum-culture conversion is linked to poorer end-of-treatment outcomes and remains a useful intermediate phenotype (Kurbatova et al., 2015; Ahmad N et al., 2018). Contemporary all-oral regimen trials have improved treatment prospects (Nyang’wa BT et al., 2022), but they also reinforce the clinical importance of matching the regimen to the resistance profile and of recognizing residual microbiological risk during treatment (García-Marín et al., 2024). A recent whole-genome sequencing (WGS) and machine-learning study of rifampicin-resistant tuberculosis (RR-TB) further highlighted delayed sputum conversion as a phenotype shaped by both clinical and genomic factors, motivating focused analyses of resistance heterogeneity (Fang et al., 2025).

WGS has improved the resolution of *M. tuberculosis* drug-resistance interpretation (García-Marín et al., 2024). Large collaborative datasets have shown that sequence-based prediction can identify susceptibility and resistance to key anti-tuberculosis drugs, while analytical frameworks such as TB-Profiler and standardized interpretation rules have made WGS results more reproducible across settings (Coll et al., 2015; Meehan et al., 2019). WHO mutation catalogues have further formalized how genomic variants should be interpreted for clinical and public-health use, including the evidence grading of mutations associated with resistance to first-line, second-line, and newer drugs (Miotto et al., 2017; Walker et al., 2022). However, most routine WGS interpretation still focuses on consensus-level or fixed mutations. This approach is clinically sensible because fixed mutations are easier to validate and standardize, but it leaves a potential blind spot: resistant subpopulations can exist below the consensus threshold and may become relevant under drug pressure.

Minority variants and heteroresistance are biologically plausible contributors to delayed treatment response, but their interpretation is difficult (Engelthaler et al., 2019). Low-frequency variants may indicate within-host microevolution, mixed infection, emerging resistance, lineage-linked diversity, or technical artefacts introduced during sequencing, mapping, or variant calling (Goossens et al., 2022; Zhang Xiaomei et al., 2023). Several studies have linked phenotypic heterogeneity, within-host diversity, or serial within-host evolutionary change with poor early response, drug resistance emergence, or complicated transmission histories (Zetola NM et al., 2014; Cohen T et al., 2016; Trauner et al., 2017). At the same time, methodological work has shown that detecting minority variants in *M. tuberculosis* WGS data depends strongly on depth, quality thresholds, allele-frequency cutoffs, and variant-calling strategy (Goossens et al., 2022). Thus, the core clinical question is not simply whether minority variants are present, but whether they occur in a treatment-relevant genetic context.

We therefore considered a minority variant through three linked conditions: the fixed-resistance background, the affected resistance gene, and whether that gene corresponded to a drug actually administered. This framework differs from a burden-only approach. A low-frequency variant in an unused drug target may have little bearing on treatment response, whereas a higher-frequency minority variant in a target of an active regimen could mark a subpopulation exposed to relevant selection pressure (Zhang Xiaomei et al., 2023). In RR/MDR-TB, where regimens often combine drugs with variable potency, tolerability, and phenotypic drug-susceptibility testing (pDST) support, this distinction may be clinically important but requires cautious evaluation (Ahmad N et al., 2018; Conradie F et al., 2022).

To address this question, we analysed a citywide RR/MDR-TB cohort from Ningbo, China, where patients were diagnosed and managed through the municipal tuberculosis care network, including designated hospitals and district-level TB dispensaries or clinics. Clinical records, phenotypic drug-susceptibility testing, treatment exposure during the first 6 months, microbiological follow-up, and WGS-derived fixed and minority variants were available for integrated analysis. Our primary analyses focused on classic tuberculosis drug-resistance genes, with the broader WHO catalogue gene set used as a sensitivity analysis. We asked whether minority variants were more informative when they occurred at higher allele frequencies and involved genes targeted by drugs actually administered, rather than treating overall minority-variant burden as a single undifferentiated measure.

## Methods

### Study design and cohort

This retrospective study included culture-positive RR-TB patients diagnosed and managed through the citywide tuberculosis care network in Ningbo, China, including designated hospitals and district-level TB dispensaries or clinics, from Jan 1, 2021, to Dec 31, 2025. For patients who received treatment in Ningbo, culture-positive isolates were submitted to the Ningbo Municipal Center for Disease Control and Prevention (Ningbo CDC) for phenotypic drug-susceptibility testing. The analysis included patients who received a programmatic RR-TB regimen and had documented treatment records and microbiological follow-up. Patients were excluded if they were pregnant, infected with human immunodeficiency virus (HIV), hepatitis B virus, or hepatitis C virus, or refused to participate. Demographic, clinical, and microbiological records were retrieved from the national TB information management system. The study was approved by the Ethics Committee of the Ningbo Municipal Center for Disease Control and Prevention (Approval No. 202314). Written informed consent was obtained from all participants.

### Outcomes

The primary microbiological outcome was sputum culture conversion, defined according to WHO recommendations as two consecutive negative cultures collected at least 30 days apart (Mirzayev et al., 2021). Culture status was assessed at 2 and 6 months after treatment initiation. Positivity at each time point was analysed as a binary outcome, and delayed culture conversion was defined as positivity at either assessment. Patients with missing culture results were excluded only from the corresponding outcome-specific model. The clinical outcome was unfavourable treatment outcome, defined according to WHO criteria as death, treatment failure, versus favourable outcome (cure or treatment completion).

### Phenotypic drug susceptibility testing

Phenotypic drug susceptibility tests of four first-line anti-TB drugs and five second-line drugs were conducted on solid media (Löwenstein-Jensen) based on WHO recommendations (World Health Organization, 2018). The drug concentrations were as follows: isoniazid (INH) 0.2 μg/ml, rifampicin (RIF) 40 μg/ml, ethambutol (EMB) 2 μg/ml, streptomycin (SM) 4 μg/ml, levofloxacin (LVX) 2 μg/ml, amikacin (AM) 30 μg/ml, capreomycin (CM) 40 μg/ml, prothionamide (PTO) 40 μg/ml, and para-aminosalicylic acid (PAS) 1 μg/ml. H37Rv strains were used as a reference for quality control. All pDST procedures were performed in a single reference laboratory using standardized protocols.

### WGS and data preprocessing

*M. tuberculosis* culture products were inactivated by heat treatment at 80 °C for 20 min (Sabiiti et al., 2019), and genomic DNA was isolated using a bacterial DNA extraction kit (QIAGEN Inc., Dusseldorf, Germany), according to the manufacturer’s instructions. The purified genomic DNA was quantified using a TBS-380 fluorometer (Turner BioSystems Inc., Sunnyvale, CA, USA) before library preparation. At least 1 μg of genomic DNA per sample was used as input. DNA was fragmented to approximately 400 bp, and libraries were generated using the NEXTflex Rapid DNA-Seq Kit. Libraries were sequenced on an Illumina NovaSeq 6000 platform using paired-end 150 bp reads.

Raw sequencing data were processed using fastp (v0.20.1) (Chen et al., 2018). Adapter sequences were trimmed, reads with ≥50% bases having Phred quality scores < 20 were discarded, and the 3′ ends of reads were trimmed to a quality threshold of Q20. Reads shorter than 50 bp after trimming were removed. Paired reads in which one mate failed quality filtering were both discarded. High-quality sequence data were then input into Kraken2 for species identification using the Standard database, and samples identified as other species or with an *M. tuberculosis* complex proportion below 90% were rejected as contaminated samples (Wood et al., 2019). Samples with evidence of mixed-strain infection were identified using TBtypeR (v25.01.1) and excluded from all downstream analyses to avoid confounding from polyclonal allelic variation (Munro et al., 2025).

### WGS-based variant detection and annotation

Clean reads passing quality control were aligned to the *M. tuberculosis* H37Rv reference genome (RefSeq: NC_000962.3) using BWA-MEM (v0.7.17) with default parameters (Li, 2013). Fixed single-nucleotide polymorphisms (SNPs) were called using FreeBayes (v1.3.10) with a minimum alternate AF threshold of 95% (Garrison and Marth, 2012). Minority variants were detected using LoFreq (v2.1.5) with a minimum AF threshold of 1% (Wilm A et al., 2012). Variants from both callers were subsequently filtered using BCFtools (v1.20) to retain only variants with site quality (QUAL) > 30 (Danecek et al., 2021). Minority variants were additionally required to have an alternate-read depth ≥ 10, an allele-specific strand balance (forward-read fraction) between 0.10 and 0.90, and a strand-bias Phred score ≤ 20. Treatment-matched AF ≥ 5% minority RAVs were verified by manual inspection of read alignments in the Integrative Genomics Viewer (IGV) v2.19.4 (Robinson et al., 2011), applying four pre-specified criteria: ≥ 10 alternate-supporting reads, biallelic strand support, clean flanking sequences, and no soft-clip clusters. Both fixed and minority variants were filtered using tb_variant_filter (https://github.com/COMBAT-TB/tb_variant_filter) to exclude variants in low-mappability regions defined by Refined Low Confidence (RLC) and RLC-plus-low-mappability regions from Marin et al (Marin et al., 2022; van Heusden et al., 2022).

Drug resistance-associated mutations were identified by inputting both filtered fixed and minority variants into TB-Profiler (v6.5.0) against the WHO V2 mutation catalogue, which classifies mutations into tiers including “Assoc w R” and “Assoc w R – Interim” (Walker et al., 2022; World Health Organization, 2023). Putative compensatory mutations were identified using the CompMut-TB framework, which applies association and mediation testing on large-scale WGS data to detect mutations that likely offset the fitness cost of primary drug resistance (Billows et al., 2024). Detected variants were annotated using SnpEff (v5.3.0), and annotated fixed and minority variants were retained for downstream analyses (Cingolani et al., 2012). Phylogenetic lineages and sublineages were assigned using TB-Profiler based on canonical lineage-defining SNPs (Coll et al., 2015).

### Phylogenetic reconstruction

Phylogenetic analysis was performed by concatenating fixed SNPs into a multi-sequence alignment. Maximum-likelihood (ML) phylogenetic trees were inferred using IQ-TREE v2 with the parameters “-m TEST -B 1000”, specifying automatic model selection via ModelFinder and 1, 000 ultrafast bootstrap replicates (Minh et al., 2020). The best-supported ML tree was rooted using *M. canettii* (RefSeq: NC_015848.1) as the outgroup. The resulting phylogenetic tree was visualized using the Interactive Tree of Life (iTOL) website (Letunic and Bork, 2021).

### Resistance-gene panel and variant features

The primary analyses used a predefined classic drug-resistance gene panel spanning core first-line, second-line, and newer or repurposed anti-tuberculosis drugs (40 genes: *katG*, *inhA*, *fabG1*, *ahpC*, *kasA*, *ndh*, *pncA*, *embA*, *embB*, *embC*, *ubiA*, *rpoB*, *rpoC*, *gyrA*, *gyrB*, *rrs*, *eis*, *rpsL*, *gidB*, *tlyA*, *ethA*, *thyA*, *folC*, *alr*, *ddl*, *ald*, *cycA*, *atpE*, *rv0678*, *pepQ*, *mmpL5*, *mmpS5*, *rplC*, *rrl*, *ddn*, *fgd1*, *fbiA*, *fbiB*, *fbiC*, *fbiD*). **Supplementary Table S1** lists the genes, corresponding drugs or drug classes, and gene-level variant counts. Fixed resistance-associated variant (RAV) burden was calculated as the count of consensus-level variants matching WHO V2 mutation catalogue entries (confidence tiers “Assoc w R” and “Assoc w R – Interim”) within this panel (World Health Organization, 2023). Loss-of-function variants in catalogue resistance genes not themselves listed in the catalogue (frameshift, stop-gained, start-lost) were tracked separately as novel LoF candidates but were not counted as RAVs. Minority RAVs were defined as LoFreq-detected low-frequency variants matching the same WHO V2 catalogue entries at AF ≥ 3%, the approximate LoFreq detection limit; higher-frequency subsets were defined at AF ≥ 5% and AF ≥ 10%. Treatment-matched minority RAVs were variants occurring in genes linked to drugs administered during the first 6 months of treatment. To avoid misclassifying low-coverage isolates as variant-free, isolates rather than variant calls were excluded: patients whose target-gene median depth fell below 200× were removed from the corresponding drug’s treatment-matched analysis, a threshold at which LoFreq detects minority variants at ≥10% allele frequency with >90% sensitivity (Goossens et al., 2022); every minority RAV retained for analysis had site depth ≥ 200×.

### Treatment exposure

Treatment exposure during the first 6 months was abstracted at the drug level. The regimen drug count was the number of anti-tuberculosis drugs recorded during this period. Because pDST was performed for the nine routinely tested drugs and did not include all newer or repurposed agents, this count was used as a treatment-exposure descriptor rather than as a fully pDST-confirmed effective-drug count. Fluoroquinolone use was defined as receipt of levofloxacin or moxifloxacin, which were treated as a single drug class. Bedaquiline and linezolid use were defined and analysed separately, as receipt of bedaquiline and receipt of linezolid, respectively. Treatment-target mapping was restricted to patients for whom administered drugs could be linked to predefined resistance genes. Because WGS was performed on isolates obtained at the time of diagnosis, generally before treatment initiation, treatment-matched minority RAVs represent resistant subpopulations present in the baseline isolate for which the linked drug was administered during the first 6 months, although isolates collected after treatment start in a minority of patients cannot fully exclude variants that emerged after drug exposure.

### Statistical analysis

We summarised fixed and minority variant burden, AF distributions, treatment-matched variant profiles, pDST resistance burden, treatment exposure, and microbiological outcomes as median (interquartile range [IQR]) or frequency (percentage), as appropriate. Denominators were reported according to data availability. Patients with missing culture results were excluded only from the corresponding outcome-specific analysis; regression models used complete cases for the outcome, exposure, and covariates included. Treatment-matched analyses were restricted to patients for whom drugs received during the first 6 months could be mapped to predefined target genes.

Logistic regression was used to evaluate associations between fixed resistance-gene burden, minority-variant features, and microbiological outcomes. Models were adjusted for age, sex, diabetes, baseline smear status, treatment history, and major lineage; minority-variant models were additionally adjusted for fixed resistance-gene burden. To limit overfitting in this exploratory cohort, models were kept parsimonious; cavitary disease was accordingly examined in a sensitivity analysis rather than the primary covariate set, and the main estimates were unchanged after its inclusion. Estimates from sparse subgroups, particularly treatment-matched AF ≥ 5% minority-variant carriers, were interpreted cautiously.

Odds ratios (ORs), adjusted odds ratios (aORs), 95% profile-likelihood confidence intervals (CIs), nominal p values, and Benjamini-Hochberg false-discovery-rate (FDR) q values were reported. FDR correction was applied within prespecified analysis families, such as outcome-specific fixed-burden and minority-variant model sets, gene-level screens, and sensitivity analyses. Discriminative performance was assessed by comparing area under the receiver operating characteristic curve (AUC) values between a baseline model containing clinical covariates and fixed resistance-gene burden, and extended models incorporating minority-variant features, with significance evaluated by likelihood-ratio tests. Analyses and figures were generated in R using the tidyverse and ggplot2 packages, and receiver operating characteristic analyses were conducted with the pROC package (Robin et al., 2011; Villanueva and Chen, 2019; Wickham et al., 2019).

## Results

### Analytic cohort and genomic variant landscape

From 2021 to 2025, 5, 232 patients had positive sputum cultures, and 328 patients with RR-TB were assessed for eligibility (**Figure 1A**). Baseline clinical, microbiological, treatment, and genomic characteristics are summarised in **Table 1**. Sixty-eight patients were excluded before treatment because they were lost to follow-up (*n* = 19), refused treatment (*n* = 24), died before treatment (*n* = 4), or transferred out (*n* = 21). Of the 260 patients who received a programmatic RR-TB regimen, 31 were excluded during genomic quality control or species confirmation: 19 DNA-extraction failures, 6 contaminated strains, 5 non-tuberculous mycobacterial isolates, and one TBtypeR-defined mixed-infection sample. The final analytic cohort comprised 229 patients. Culture positivity was recorded in 86/229 patients (37.6%) at 2 months and 56/229 (24.5%) at 6 months. Final treatment outcome was determined for 201 patients after excluding 28 still receiving treatment; 131 had favorable outcomes and 70 had unfavorable outcomes.

**Figure 1:**
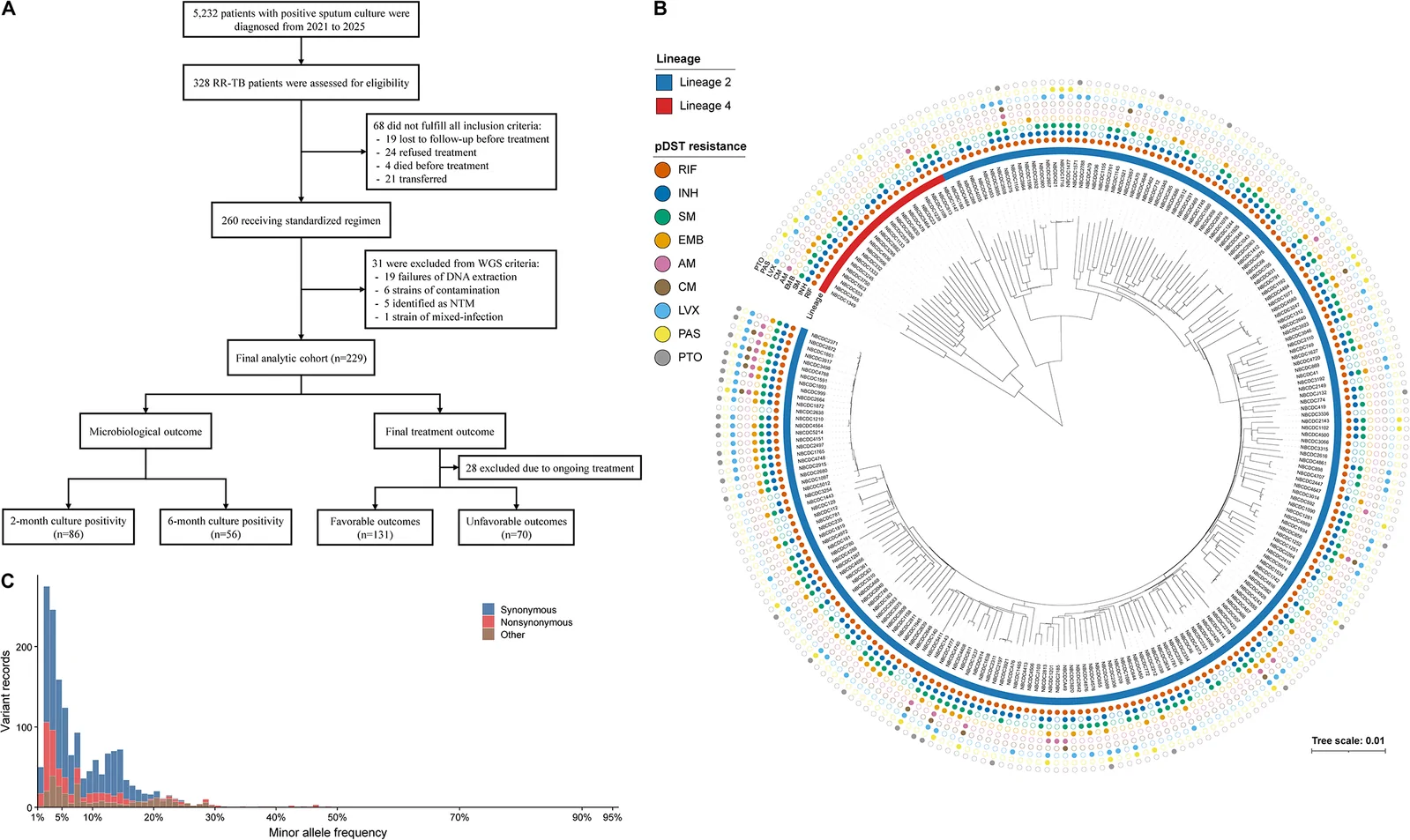
Study cohort, strain phylogeny, and resistance-gene minority variation. **(A)** Patient eligibility, WGS-based exclusions, TBtypeR mixed-infection quality control, and final cohort assembly. **(B)** Maximum-likelihood phylogenetic tree of the final 229 isolates. The colour strip indicates major *M. tuberculosis* lineages, and outer annotation rings show pDST profiles for RIF, INH, SM, EMB, AM, CM, LVX, PAS, and PTO. Filled circles indicate phenotypic resistance, and empty circles indicate phenotypic susceptibility. The scale bar shows the number of substitutions per site. **(C)** AF ≥ 1% minority-variant frequency distribution within classic drug-resistance genes, stratified by annotated effect class.

**Table 1:** Baseline clinical, microbiological, treatment, and genomic characteristics of the analytic cohort by 6-month culture status.

Section	Characteristic	Overall (n *=* 229)	6-month culture negative (n = 173)	6-month culture positive (n = *5*6)
Demographic and clinical characteristics	Age, years, median (IQR)	45 (31-58)	43 (31-58)	46 (32-56)
	Male sex	178/229 (77.7%)	132/173 (76.3%)	46/56 (82.1%)
	Diabetes	48/229 (21.0%)	42/173 (24.3%)	6/56 (10.7%)
	Cavitary disease	128/219 (58.4%)	97/168 (57.7%)	31/51 (60.8%)
	Previously treated TB	93/226 (41.2%)	65/170 (38.2%)	28/56 (50.0%)
	Baseline smear positive	118/229 (51.5%)	83/173 (48.0%)	35/56 (62.5%)
Lineage	Lineage 2	206/229 (90.0%)	154/173 (89.0%)	52/56 (92.9%)
pDST	INH resistant	161/229 (70.3%)	116/173 (67.1%)	45/56 (80.4%)
	SM resistant	131/229 (57.2%)	98/173 (56.6%)	33/56 (58.9%)
	EMB resistant	74/229 (32.3%)	52/173 (30.1%)	22/56 (39.3%)
	LVX resistant	72/229 (31.4%)	48/173 (27.7%)	24/56 (42.9%)
	AM resistant	22/229 (9.6%)	12/173 (6.9%)	10/56 (17.9%)
	CM resistant	14/229 (6.1%)	8/173 (4.6%)	6/56 (10.7%)
	PAS resistant	28/229 (12.2%)	17/173 (9.8%)	11/56 (19.6%)
	PTO resistant	32/229 (14.0%)	22/173 (12.7%)	10/56 (17.9%)
	RIF and INH resistant	161/229 (70.3%)	116/173 (67.1%)	45/56 (80.4%)
	RIF and LVX resistant	72/229 (31.4%)	48/173 (27.7%)	24/56 (42.9%)
	pDST-resistant drugs, median (IQR)	3 (2-4)	3 (2-4)	4 (3-5)
Treatment and genomic features	Drugs received in first 6 months, median (IQR)	5 (4-5)	5 (5-5)	5 (3-5)
	Fluoroquinolone used in first 6 months	155/229 (67.7%)	122/173 (70.5%)	33/56 (58.9%)
	Bedaquiline or linezolid used in first 6 months	145/229 (63.3%)	117/173 (67.6%)	28/56 (50.0%)
	Fixed RAV burden, median (IQR)	3 (2-5)	3 (2-5)	4 (3-5)
	Any AF ≥ 3% minority RAV	13/229 (5.7%)	7/173 (4.0%)	6/56 (10.7%)
	Any AF ≥ 5% minority RAV	11/229 (4.8%)	5/173 (2.9%)	6/56 (10.7%)
	Treatment-matched AF ≥ 5% minority variant	7/205 (3.4%)	3/161 (1.9%)	4/44 (9.1%)

Values are n/N (%) unless otherwise indicated. Treatment-matched minority-variant denominators are restricted to patients with interpretable first-6-month treatment-target mapping (n = 205 overall; n = 161 culture negative; n = 44 culture positive). AF, allele frequency; AM, amikacin; CM, capreomycin; EMB, ethambutol; INH, isoniazid; LVX, levofloxacin; PAS, para-aminosalicylic acid; pDST, phenotypic drug-susceptibility testing; PTO, prothionamide; RIF, rifampicin; SM, streptomycin.

Sequencing depth was high across the cohort, including the predefined drug-resistance regions used for fixed- and minority-variant analyses. The median genome-wide depth was 288.0× (IQR 248.0-329.0), and the median depth across drug-resistance target regions was 293.0× (IQR 253.0-330.0). Target-region coverage was essentially complete, with a median depth-pass proportion of 100.0% (IQR 100.0-100.0) (**Supplementary Table S2**).

Most isolates belonged to lineage 2 (206/229, 90.0%), with the Beijing-associated sublineage 2.2.1 predominating (189/229, 82.5%), followed by 2.2.2 (17/229, 7.4%). Lineage 4 accounted for 23/229 isolates (10.0%), comprising sublineages 4.5 (10/229, 4.4%), 4.4.2 (9/229, 3.9%), 4.2.2 (3/229, 1.3%), and 4.4 (1/229, 0.4%) (**Figure 1B**). pDST results were complete for all 229 isolates across the nine tested drugs. RIF resistance was present in all isolates (229/229, 100.0%); resistance to INH and SM occurred in 161/229 (70.3%) and 131/229 (57.2%) isolates, respectively. Resistance to EMB and LVX was detected in 74/229 (32.3%) and 72/229 (31.4%) isolates, respectively. Resistance to second-line injectable drugs was less common, with 22/229 (9.6%) resistant to AM, 14/229 (6.1%) resistant to CM, and 24/229 (10.5%) resistant to either AM or CM. Resistance to PAS and PTO was observed in 28/229 (12.2%) and 32/229 (14.0%) isolates, respectively. Overall, 161/229 isolates (70.3%) were phenotypically resistant to both RIF and INH, and 72/229 (31.4%) showed combined RIF and LVX resistance. The median number of pDST-resistant drugs was 3 (IQR 2-4).

Callable AF ≥ 1% minority variants were observed genome-wide in all 229 samples (**Figure 1C**). In total, 42, 488 genome-wide minority-variant records were identified, with a right-skewed per-sample burden (median 57 records, IQR 36-95) (**Supplementary Figure S1**). The median AF was 5.6% (IQR 3.1-10.5%), supported by a median site depth of 324 reads (IQR 275-364) and 17 minor-allele reads (IQR 10-29). Records were distributed across the 1-5% AF range (19, 108 [45.0%]), 5-10% range (11, 824 [27.8%]), and ≥ 10% range (11, 556 [27.2%]); 24, 135 (56.8%) were synonymous and 15, 703 (37.0%) nonsynonymous.

Within classic drug-resistance genes, 846 fixed RAV records were identified across 229 patients, with 220 (96.1%) carrying at least one fixed RAV (median 3 per patient, IQR 2–5). Thirty-two minority RAV records were detected across 16 patients; 28 records (13 patients) met the AF ≥ 3% threshold, of which 22 (11 patients) met the AF ≥ 5% threshold. Four RAV records below the AF ≥ 3% detection limit were excluded. Each minority RAV was annotated with site depth, ALT-supporting reads, forward/reverse read counts, and strand balance, with all sites showing depth ≥ 200× (range 214–405×). Per-variant depth and AF distributions for all minority RAVs are shown in **Supplementary Figure S2**. All 14 treatment-matched AF ≥ 5% minority RAVs were manually inspected and confirmed with ≥ 10 alternate-supporting reads and biallelic strand support (**Supplementary Figure S3**; **Supplementary Table S3**). The minority RAV spectrum was biologically coherent, involving *pncA* (pyrazinamide), *gyrA* (fluoroquinolones), *embB* (ethambutol), and *rpoB* (rifampicin).

Among 205 patients evaluable after 200× coverage exclusion, treatment-matched AF ≥ 5% minority RAVs were present in 7 patients. Because drug receipt does not necessarily reflect effective drug pressure, we audited each treatment-matched minority RAV against baseline pDST results and same-drug fixed RAVs (**Supplementary Table S4**). All 14 treatment-matched AF ≥ 5% minority RAVs occurred in isolates with pre-existing resistance evidence: baseline pDST resistance to the linked drug (6/14), microheterogeneity with the same variant called at majority level (3/14), or another fixed RAV for the same drug (5/14); none arose in an isolate without concurrent evidence of drug resistance.

The coverage exclusion primarily affected linezolid (66/132 excluded) and aminoglycoside targets (13/33 for amikacin) due to systematically low *rrl*/*rrs* depth. In a more stringent sensitivity analysis, any isolate–drug pair in which any site within the target gene had depth < 200× was excluded; 171 patients remained evaluable and four treatment-matched AF ≥ 5% carriers were retained. The association with 6-month culture positivity remained in the same direction but was no longer significant (aOR 5.29, 95% CI 0.57–49.02; *p* = 0.143), reflecting reduced power from the smaller carrier group. Fifteen novel LoF candidates outside the catalogue were identified in genes including embA, ald, and ubiA, and were tracked separately as potential resistance markers pending functional validation. The complete variant catalogue, per-variant QC metrics, treatment-matched audit, target gene coverage summary, and novel LoF candidates are provided in **Supplementary Table S2**.

### Adjusted genomic associations with microbiological response

In clinically adjusted models, fixed RAV burden showed a borderline nominal association with delayed culture conversion (defined as culture positivity at month 2 or 6; aOR 1.13, 95% CI 1.00–1.27; p = 0.044; q = 0.178) that did not survive multiple-testing correction, and no clear association with 2-month culture positivity (aOR 1.10, 95% CI 0.98–1.24; p = 0.097), 6-month culture positivity (aOR 1.07, 95% CI 0.94–1.21; *p* = 0.327), or unfavourable outcome (aOR 0.99, 95% CI 0.87–1.13; *p* = 0.859) (**Supplementary Figure S4**). Fixed RAV burden was therefore included as an adjustment covariate rather than a standalone predictor in all minority-variant models.

Minority RAVs showed threshold-dependent adjusted associations with 6-month culture positivity when analysed without treatment context. Any minority RAV at AF ≥ 3% showed a borderline association after adjustment for fixed RAV burden and clinical covariates (aOR 3.24, 95% CI 0.94–11.12; *p* = 0.057; *q* = 0.071), which became significant at AF ≥ 5% (aOR 5.53, 95% CI 1.46–22.97; *p* = 0.013; *q* = 0.032). The clearest signal involved treatment-matched AF ≥ 5% minority RAVs and 6-month culture positivity (aOR 7.21, 95% CI 1.38–43.68; *p* = 0.020; *q* = 0.034), confirmed by Firth penalized regression (OR 6.23, 95% CI 1.20–32.28; *p* = 0.029; **Supplementary Table S5**). The association was robust to further adjustment for cavitary disease (OR 6.19, 95% CI 1.17–32.63; p = 0.032; **Supplementary Table S5**), which was balanced across outcome groups (60.8% vs 57.7%). Event-rate contrasts were consistent across thresholds: for treatment-matched AF ≥ 3% minority RAVs, 44.4% (4/9) of carriers were culture-positive at 6 months versus 20.4% (40/196) of non-carriers; for AF ≥ 5%, the corresponding contrast was 57.1% (4/7) versus 20.2% (40/198). This association was specific to the 6-month endpoint, with no clear signal for 2-month culture positivity (aOR 3.13, 95% CI 0.63–17.45; *p* = 0.16) or unfavourable outcome (aOR 1.37, 95% CI 0.18–7.25; *p* = 0.72).

### Gene-level signals within the resistance panel

Exploratory gene-level Fisher tests, restricted to consensus-level WHO V2 catalogue RAVs with at least five carriers, identified *pncA* as the dominant fixed signal (**Figure 2**). Fixed *pncA* RAVs, carried by 68/229 patients, were associated with 2-month culture positivity after FDR correction (37/68 [54.4%] vs 49/161 [30.4%]; OR 2.72, 95% CI 1.46–5.10; *p* = 0.0009; *q* = 0.010) and with delayed culture conversion (39/68 [57.4%] vs 55/161 [34.2%]; OR 2.59, 95% CI 1.39–4.83; *p* = 0.0019; *q* = 0.021), with a nominal trend towards 6-month culture positivity (24/68 [35.3%] vs 32/161 [19.9%]; OR 2.19, 95% CI 1.11–4.31; *p* = 0.018; *q* = 0.199). No other fixed RAV gene showed a significant association with any outcome after FDR correction, and the small number of minority RAV carriers (13 patients) precluded meaningful gene-level minority-variant screening. As these gene-level tests were unadjusted screening analyses, they are best regarded as hypothesis-generating, and any independent predictive role for individual genes remains to be confirmed in covariate-adjusted models.

**Figure 2:**
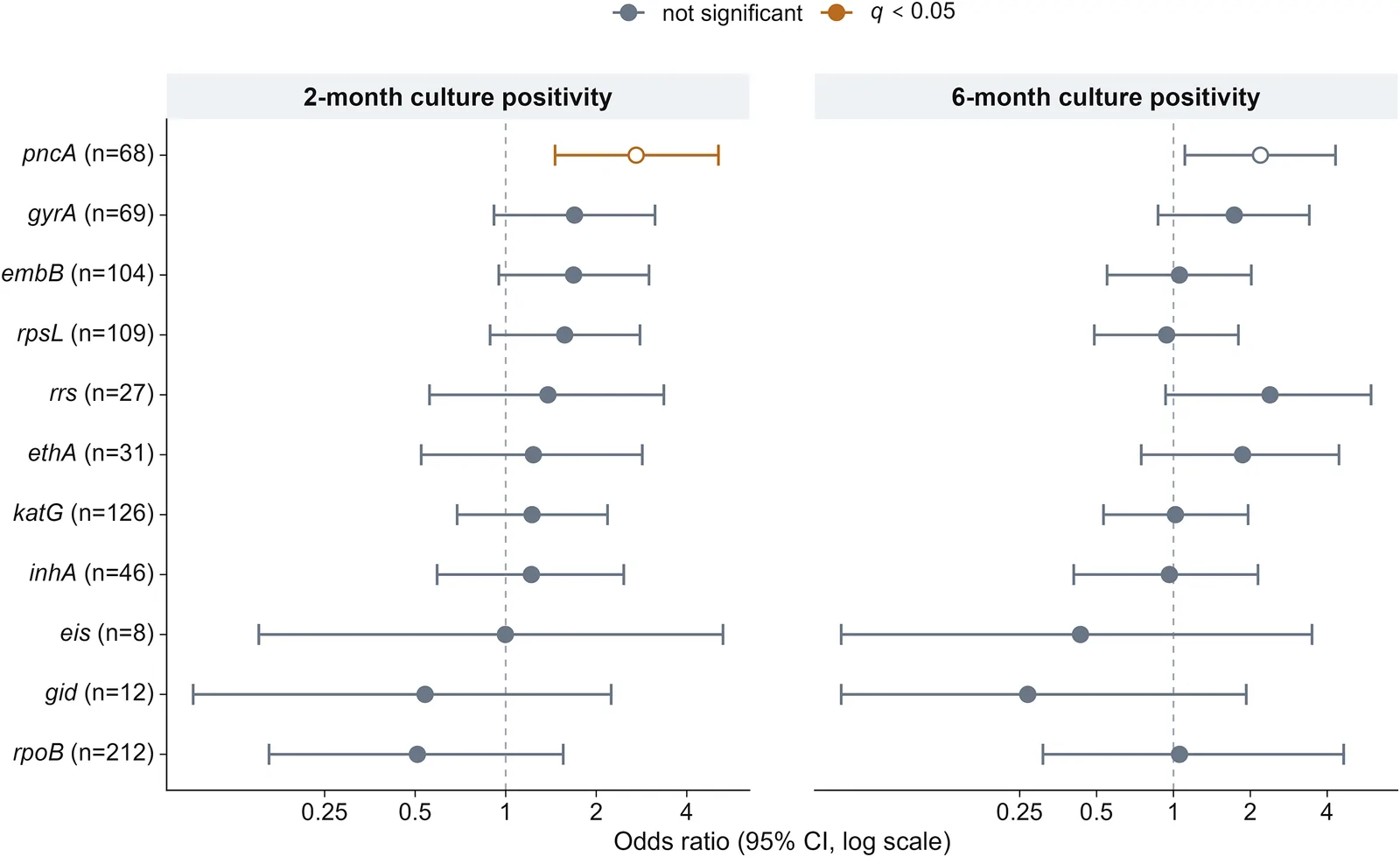
Gene-level fixed RAV signals within the resistance panel. Exploratory gene-level Fisher tests, restricted to consensus-level WHO V2 catalogue RAVs with at least five carriers, are shown for 2-month and 6-month culture positivity. Points indicate odds ratios and horizontal lines 95% confidence intervals (log scale); terracotta points indicate FDR-significant associations (*q* < 0.05). Numbers in parentheses indicate carriers per gene.

### Minority variants added limited predictive information

Minority-variant features added limited predictive information (**Supplementary Figure S5**). For 6-month culture positivity, adding treatment-matched AF ≥ 5% minority variants increased the apparent AUC from 0.675 to 0.695 in the treatment-matched subset (**Figure 3**; ΔAUC 0.021; likelihood-ratio p = 0.020; *q* = 0.034; **Supplementary Table S6**). The increment was small and is not presented as a clinically deployable prediction model but supports the interpretive value of treatment-matched minority RAVs beyond fixed RAV burden and clinical covariates.

**Figure 3:**
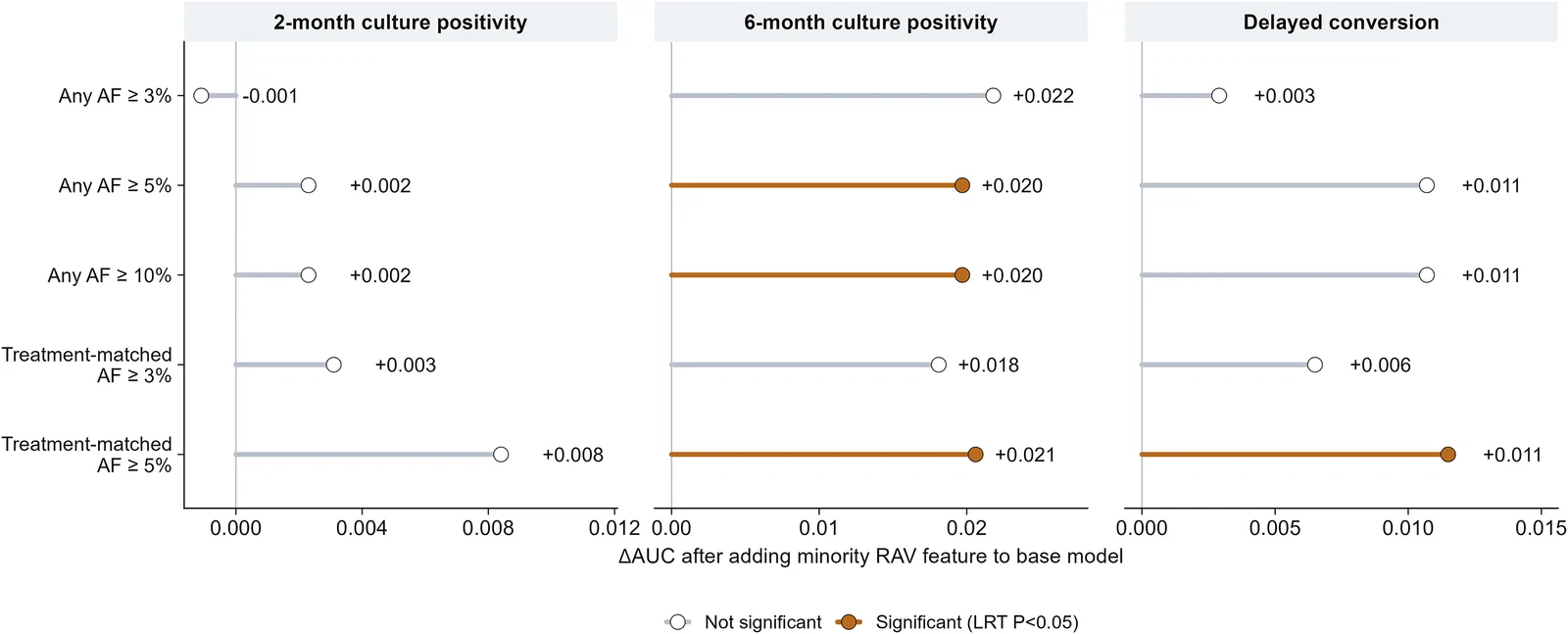
Incremental apparent AUC for 6-month culture positivity. Bars show the AUC of clinical covariates plus fixed RAV burden (baseline) and after adding treatment-matched AF ≥ 5% minority RAVs (0.675 to 0.695; ΔAUC 0.021; likelihood-ratio *p* = 0.020; *q* = 0.034).

### Resistance background and sensitivity analyses

We next examined treatment-matched AF ≥ 5% minority RAVs together with fixed RAV burden, dichotomised at the cohort median (≤3 vs ≥4 RAVs; **Figure 4**). Among patients without treatment-matched AF ≥ 5% minority RAVs, 6-month culture positivity was higher in the high fixed-RAV-burden group than in the low-burden group (25/92 [27.2%] vs 15/106 [14.2%]). The highest event rate was observed among patients with both high fixed RAV burden and treatment-matched AF ≥ 5% minority RAVs (4/5 [80.0%]). The low-burden carrier group was small (*n* = 2) with no events, limiting interpretation of this stratum.

**Figure 4:**
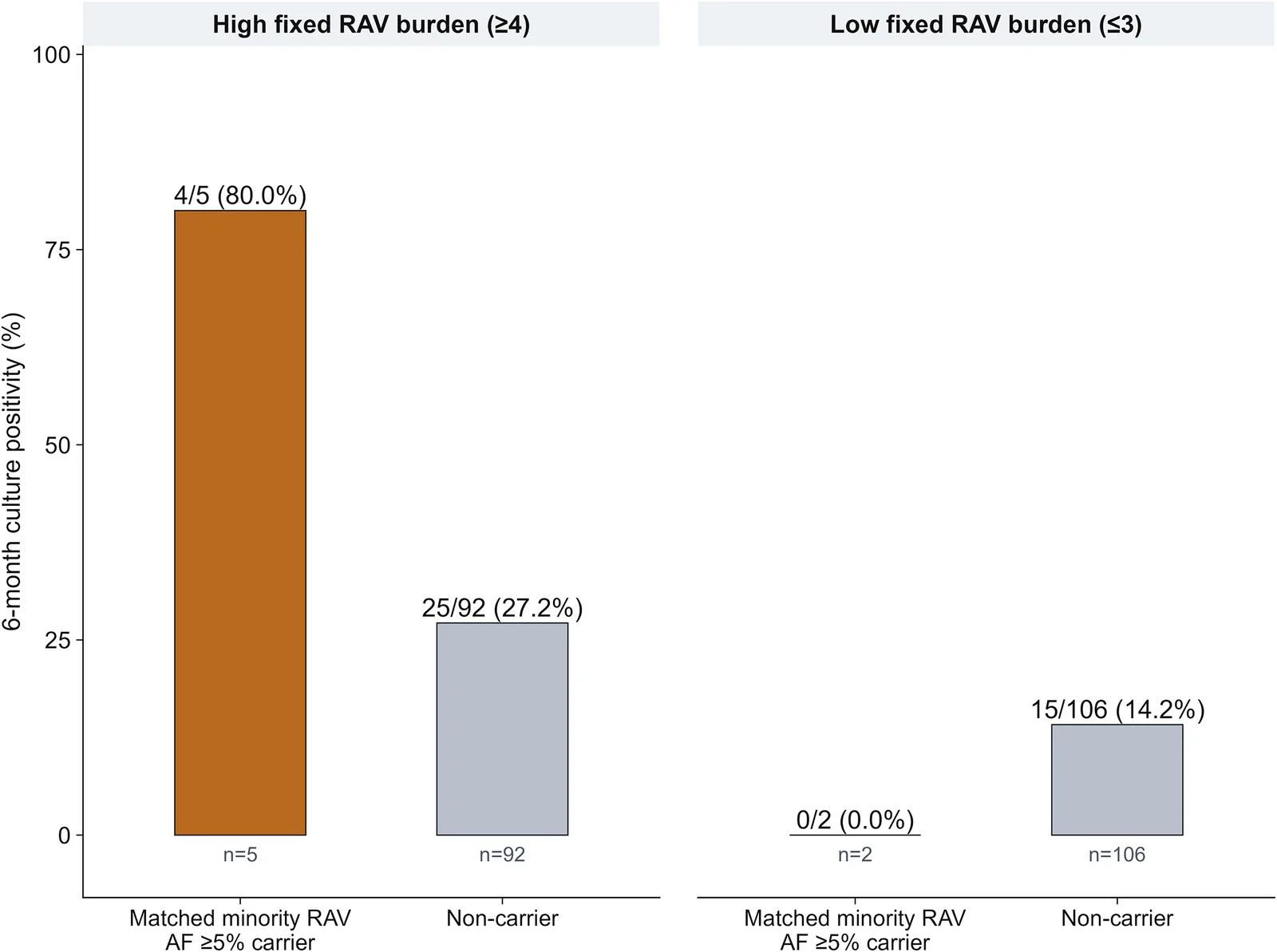
Six-month culture positivity by fixed RAV burden and treatment-matched minority-RAV status. Fixed RAV burden (WHO V2 catalogue-annotated resistance-associated variants) was dichotomised at the cohort median (≤3 vs ≥4 RAVs per patient). Treatment-matched minority RAVs were defined as AF ≥ 5% WHO V2 catalogue RAVs in genes targeted by drugs received during the first 6 months; analyses were restricted to 205 patients with interpretable drug–gene coverage (target gene median depth ≥ 200×). Cell colour denotes the crude proportion of patients with 6-month culture positivity, and cell labels show percentage and events/total.

A sensitivity analysis using the broader WHO catalogue gene set increased minority-variant detection but did not strengthen the treatment-matched association (**Supplementary Table S7**). This expanded definition captured 3, 744 AF ≥ 1% minority-variant records across 122 patients, and 30/229 patients carried treatment-matched AF ≥ 5% minority variants. In the clinically adjusted 6-month model, the association between treatment-matched AF ≥ 5% minority variants and culture positivity was weaker than in the classic-gene analysis (aOR 1.68, 95% CI 0.65-4.13; *p* = 0.268; *q* = 0.912). Replicating the original broader definition on the current analytic cohort (any variant in resistance genes, AF ≥ 5%) yielded an attenuated association (aOR 2.47, 95% CI 0.86–7.13; p = 0.094; 19 carriers), consistent with the re-defined exposure.

Conversely, as a specificity control, we examined minority variants in resistance genes that were not classified as resistance-associated in the WHO V2 catalogue. Such non-catalogue variants were uncommon (4/229 carriers at AF ≥ 3%) and showed no association with any outcome (all p ≥ 0.57, Fisher’s exact test), with no 6-month culture-positive events among carriers; the treatment-matched AF ≥ 5% subset comprised a single patient, precluding meaningful evaluation. The outcome signal in this cohort was therefore confined to catalogue-defined resistance-associated variants.

Exploratory compensatory-variant analyses did not materially strengthen the primary findings. Among *rpoB*-mutant samples, *rpoC*/*rpoA* minority candidates showed only a nominal association with 6-month culture positivity (aOR 3.82, 95% CI 1.21–12.08; p = 0.023; q = 0.090), while literature-supported CompMut-TB matches showed no association (**Supplementary Table S5**).

## Discussion

In this Ningbo RR/MDR-TB cohort, the clinical signal from minority variants was not driven by overall burden. The clearest pattern appeared when variants were higher-frequency, treatment-matched, and assessed against fixed RAV burden: the association with 6-month culture positivity withstood FDR correction (aOR 7.21, 95% CI 1.38–43.68; *q* = 0.034), although it was estimated from only seven carriers with wide confidence intervals, and the AUC gain was modest (0.021). These results support an interpretive role for selected minority variants, not a standalone predictive marker.

One way to read this pattern is through treatment pressure, while recognizing that the data do not prove clonal selection. Consensus-level WGS remains the most reproducible basis for resistance interpretation, especially for well-validated fixed mutations (Meehan et al., 2019). Minority variants are harder to interpret because they may reflect emerging resistance, neutral diversity, mapping artefacts, mixed infection, or variation unrelated to the regimen (Engelthaler et al., 2019; Castro et al., 2021; Zhang Xiaomei et al., 2023). Linking them to administered drugs makes the signal more plausible, because only drug-exposed targets would be expected to experience relevant selection pressure.

The stronger association with 6-month culture positivity than with 2-month positivity is clinically plausible. Early culture positivity can reflect baseline bacillary burden, cavitary disease, initial regimen potency, adherence, drug absorption, and other host or treatment factors. By contrast, persistent culture positivity at 6 months is closer to sustained microbiological non-response and has been associated with poorer multidrug-resistant tuberculosis outcomes (Kurbatova et al., 2015; Ahmad N et al., 2018). A resistant subpopulation present below the consensus threshold may require sustained drug exposure before becoming clinically visible (Castro et al., 2021).

Patients with both high fixed RAV burden and treatment-matched AF ≥ 5% minority RAVs had the highest crude 6-month positivity rate (4/5 [80.0%] vs 15/106 [14.2%] in the low-burden non-carrier reference; Fisher’s exact p = 0.003), suggesting that low-frequency resistance-associated variation may be easier to interpret against a complex fixed resistance background (Zhang Xiaomei et al., 2023). The small low-burden carrier group limits interpretation of that stratum.

The results sit alongside earlier work on heteroresistance and within-host diversity, rather than replacing established resistance interpretation. Phenotypic heterogeneity, within-host diversity, and serial within-host evolutionary change have been linked to poor early response or treatment dynamics (Trauner et al., 2017; Castro et al., 2021). Minority-variant calls also remain sensitive to sequencing depth, AF threshold, strand bias, mapping uncertainty, and mixed infection (Goossens et al., 2022). In practical terms, low-frequency calls may deserve closer review when they occur at higher AF, match drugs actually used, and arise on a high fixed resistance background.

The gene-level and compensatory-variant analyses also support a cautious reading. The fixed *pncA* signal was biologically plausible but arose from an unadjusted screening analysis with multiple comparisons, and no other gene-level RAV remained significant after FDR correction. Although *rpoC*/*rpoA* compensatory mutations can mitigate fitness costs and have been linked to MDR-TB transmission (Che et al., 2025), neither CompMut-TB matches nor *rpoC*/*rpoA* minority candidates showed FDR-significant associations with 6-month culture positivity, arguing against treating compensatory variants as short-term risk markers (Billows et al., 2024).

The WHO catalogue sensitivity analysis shows the trade-off between broader detection and interpretability. Expanding the search increased minority-variant detection but did not strengthen the treatment-matched association, suggesting that outcome-association analyses may need hypothesis-specific feature definitions when low-frequency variants are numerous, heterogeneous, and not all relevant to the drugs received (Castro et al., 2021).

Several limitations frame these findings. This was a single-cohort retrospective analysis with sparse key subgroups: seven treatment-matched AF ≥ 5% carriers and five patients in the joint high-burden/treatment-matched group, with no events in the low-burden carrier stratum (*n* = 2). Estimates were imprecise, and although the treatment-matched association withstood FDR correction (*q* = 0.034), it was derived from only seven carriers, four of whom had isolates collected more than 7 days after treatment initiation. Treatment exposure was recorded at the level of drug receipt, which served as a proxy for drug pressure but may not reflect the true activity of each drug in individual patients. First-6-month treatment exposure did not fully capture drug timing, dose changes, interruptions, adherence, absorption, or adverse-event-driven regimen changes. The lineage 2-dominant cohort and limited pDST panel also constrain transferability and treatment-target mapping, and the minimal sublineage variation limits effective adjustment for residual population-structure confounding (Farooq et al., 2021; Atavliyeva et al., 2024). Minority-variant calling remains another source of uncertainty. Sequencing depth and strand-bias summaries were reassuring, but low-frequency calls remain sensitive to pipeline choices, local mapping artefacts, and residual within-sample complexity. Prospective studies should combine external cohorts with replicate or targeted deep sequencing, longitudinal sampling, detailed drug-exposure data, and standardized pDST.

## Conclusions

In this Ningbo RR/MDR-TB cohort, minority resistance-associated variants in classic drug-resistance genes were uncommon but biologically coherent, and their clinical relevance depended on treatment context rather than overall burden. While minority-RAV burden as a whole offered limited prognostic value, treatment-matched higher-frequency minority RAVs were associated with persistent 6-month culture positivity and were most informative against a background of high fixed RAV burden. This pattern is consistent with a resistant subpopulation present below the consensus threshold that may become clinically visible under sustained drug pressure, and argues for interpreting minority variants in relation to the regimen actually administered. These findings support a cautious, treatment-aware review of selected minority variants, with careful quality control, manual inspection, and external validation required before any minority-variant-based rule is considered in clinical management.

## Data Availability

The datasets presented in this study can be found in online repositories. The names of the repository/repositories and accession number(s) can be found below: https://ngdc.cncb.ac.cn/bioproject/browse/PRJCA018599, PRJCA018599.

## Supplementary material

Supplementary Figure 1 Genome-wide allele-frequency distribution of callable AF ≥ 1% minority variants, stratified by variant effect class.

Supplementary Figure 2 Site depth versus allele frequency for all minority RAVs. Points are coloured by treatment-matched status and shaped by AF category; the shaded region indicates depth ≥ 200× at AF ≥ 3%, and dashed lines mark the AF ≥ 3% and ≥ 5% thresholds and the 200× depth threshold. The 14 treatment-matched AF ≥ 5% minority RAVs are labelled.

Supplementary Figure 3 Manual inspection of the 14 treatment-matched AF ≥ 5% minority RAVs across seven carriers. Coverage (top), read-alignment (middle, coloured by strand), and gene tracks are shown for each locus; all sites met the pre-specified criteria of ≥ 10 alternate-supporting reads, biallelic strand support, clean flanking sequence, and no soft-clip clusters.

Supplementary Figure 4 Adjusted associations between fixed RAV burden, minority-variant features, and microbiological outcomes.

Supplementary Figure 5 Incremental apparent AUC after adding minority-variant features to clinical and fixed-burden models.

Supplementary Table 1 Classic resistance-gene panel, drug-target mapping, and gene-level variant counts.

Supplementary Table 2 Fixed and minority RAV catalogue, per-variant quality-control metrics, treatment-matched audit, target-gene coverage summary, and novel loss-of-function candidates.

Supplementary Table 3 Variant-level audit table for treatment-matched AF ≥ 5% minority variants.

Supplementary Table 4 Active drug-pressure audit: per-variant cross-check of treatment-matched minority RAVs against baseline phenotypic DST and same-drug fixed RAVs, with active-subset event rates.

Supplementary Table 5 Candidate compensatory-variant analysis, CompMut-TB literature-supported matches, Firth penalized regression, and cavitary disease sensitivity analysis.

Supplementary Table 6 Area under the receiver operating characteristic curve increment, likelihood-ratio tests, and false-discovery-rate correction results.

Supplementary Table 7 WHO catalogue-wide sensitivity analysis and supporting gene/drug maps.
